# Religion-Adapted Cognitive Behavioral Therapy: A Review and Description of Techniques

**DOI:** 10.1007/s10943-021-01345-z

**Published:** 2021-09-13

**Authors:** Marianna de Abreu Costa, Alexander Moreira-Almeida

**Affiliations:** 1 Graduate Program in Psychiatry and Behavioral Sciences, Universidade Federal Do Rio Grande Do Sul (UFRGS), Rua Ramiro Barcelos, 2350, Rio Branco, Porto Alegre, RS 90035-007 Brazil; 2 Research Center in Spirituality and Health (NUPES), School of Medicine, Universidade Federal de Juiz de Fora (UFJF), Rua José Lourenço Kelmer, s/n, São Pedro, Juiz de Fora, MG Brazil

**Keywords:** Cognitive behavioral therapy, Religion-adapted CBT, Faith-adapted CBT, Religion, Spirituality

## Abstract

Systematic reviews have shown the efficacy of religion-adapted cognitive behavioral therapy (R-CBT); however, many clinicians lack practical knowledge of these protocols. We describe here the techniques of religious adaptation to CBT that have proved effective. We selected randomized clinical trials comparing R-CBT with control conditions in clients with a diagnosis of a psychiatric disorder and extracted the information from their adapted manuals. The most frequent religious adaptations were the integration of religious content to perform cognitive restructuring, psychoeducation and motivation; engagement in religious activities such as behavioral activation, meditation, or prayer to help cognitive restructuring, using religious values and coping strategies. A description of these techniques is presented here, as well as some practical examples.

## Introduction

There is growing recognition of the need to make an effort to adapt culturally effective interventions to offer evidence-based interventions that are more humanized and focused on the individual (Kirmayer & Ban, 2013) as well as to address religiosity and spirituality in clinical practice and in psychotherapy (Moreira-Almeida et al., 2014, 2016). Spirituality is about the personal search for existential meaning, the sacred and transcendent, while religiosity refers to the organized system of beliefs, practices, and rituals which seek to bring the individual closer to the sacred or transcendent (Moreira-Almeida & Koenig, 2006). Religion-adapted cognitive behavioral therapy (R-CBT) involves the adaptation of secular CBT protocols through the use of patients’ religious content, to be more sensitive and client centered. Consistent evidence suggests religion-adapted psychotherapy protocols can be as effective (Lim et al., 2014), or even more effective than standard protocols (Anderson et al., 2015). This effect seems to be independent of the therapist's own religiosity. Interestingly, one study found the spiritually adapted protocol conducted by non-religious therapists was more effective than when conducted by religious therapists (Propst et al., 1992).

The relationship between religion and psychotherapy is relevant because religious beliefs and practices are often central to how religious clients create meaning in their lives (Pargament, 2011), implementing coping resources and developing resilience (Zimpel et al., 2015). Conversely, at times, religion can also be the cause of conflict or suffering, resulting in mental health problems (Johnson et al., 2007). The integration of religiousness and psychotherapy can further improve the therapeutic relationship, bolster adherence to treatment and improve the individual's capacity to deal with suffering (Martins & Moreira-Almeida, 2018). Moreover, research also shows that most clients want clinicians to address religious issues in their general sessions of psychiatry (Baetz et al., 2004) and psychotherapy (Post & Wade, 2009). These findings are supported by the recent Position Statement on religiosity and spirituality from the World Psychiatry Association which proposes a careful consideration of clients' religious beliefs and practices, regardless of the professional's beliefs (Moreira-Almeida et al., 2016). For these reasons, religiosity should be regularly and routinely assessed and addressed in psychotherapy (Moreira-Almeida et al., 2014, 2016).

Several recent systematic reviews of dozens of randomized controlled trials have found that religion-adapted psychotherapy is at least as effective as conventional psychotherapy for mental disorders (Anderson et al., 2015; Gonçalves et al., 2015; Hook et al., 2010; Lim et al., 2014; Post & Wade, 2009; Worthington et al., 2011). Cognitive behavioral therapy (CBT) is the most frequently investigated religion-adapted psychotherapy and possesses robust proof of efficacy (Pearce, 2016). However, translating this knowledge into the actual practice of psychotherapy can be a challenge, since it is not always self-evident which religious adaptations were made to the CBT protocols. Knowledge of these changes is essential for clinicians who want to use these evidence-based protocols in the care of their clients, as well as for researchers who want to investigate the active ingredients of R-CBT. Consistent with the need for culturally based interventions (Kirmayer & Ban, 2013), the objective of the present study is to identify and describe the changes made to CBT protocols in order to accommodate religiosity. For this study, we included papers assessing R-CBT using a randomized control trial design for psychiatric disorders, since randomized control trials are the gold standard for assessing treatment efficacy.

## Methods

Firstly, we reviewed studies included in three previous reviews on religious-adapted psychotherapy, two of which were systematic reviews (Anderson et al., 2015; Hook et al., 2010; Lim et al., 2014). We initially relied on these three reviews because they were the most recently published studies. In addition, three different reviews were considered to assemble a broader range of studies, thus reducing the chances of missing any articles prior to 2014. The studies conducted by Lim et al. (2014) and Anderson et al. (2015) were both systematic reviews, but Anderson et al. (2015) only included studies relating to anxiety and depression, while Lim et al. (2014) covered all mental disorders, except for substance use. Moreover, these studies appraised different databases. Although Hook et al. (2010) was not a systematic review, they did include studies assessing religious-adapted psychotherapy for general psychological problems such as unforgivingness, eating disorders, schizophrenia, alcoholism, anger, and marital issues.

We then performed a review of randomized clinical trials that have been published in peer-reviewed journals, comparing R-CBT with secular CBT or control conditions (e.g., waiting list, placebo, or treatment-as-usual) in clients diagnosed with a psychiatric disorder. Since the three reviews included studies up to January 2014, we performed a web search using PubMed, EMBASE, the Cochrane Library and PsycINFO databases covering the period from January 2013 to May 2019. We used the following search terms: (spirit* OR relig* OR faith*) AND (CBT OR “cognitive behavioral” OR “cognitive behavioural” OR “cognitive therapy”) AND (“clinical trial” OR “randomized controlled trial” OR “controlled clinical trial”). We chose these three terms (spirit* OR relig* OR faith*) because they cover various ways to refer to what we are calling R-CBT, e.g., faith/religious/spiritual-adapted CBT, spiritually integrated CBT, religious CBT, religious/spiritual therapies, etc. We used the terms ending with an "*" to include their derivatives; for example, religio* includes religiosity, religion, religious, etc. Once we had located all the studies satisfying our inclusion criteria, we then contacted all authors and co-authors to obtain the R-CBT manuals used in the clinical trials.

We only included randomized clinical trials since this design permits the reduction in bias when testing new treatments and is considered the gold standard for assessing the efficacy of interventions and studies assessing R-CBT in clients diagnosed with a psychiatric disorder. Exclusion criteria were as follows: participants with a medical illness, though without a psychiatric disorder; internet-based interventions; and contextual therapies (like mindfulness or acceptance and commitment therapy). Under cognitive-behavior intervention, we included all studies that referred to their interventions as techniques adapted to standard cognitive behavior techniques. Texts in languages other than English, Portuguese and Spanish were excluded.

Data concerning the description of R-CBT were extracted, as well as data about the primary mental health disorder, sample size, type of control group, and primary outcomes. Adapted CBT techniques were extracted from the published clinical trials, and the manuals were sent by the authors and co-authors. We asked for permission to cite any manuals that were not published. This study focused on the description of adapted CBT techniques.

## Results

**Fig. 1:**
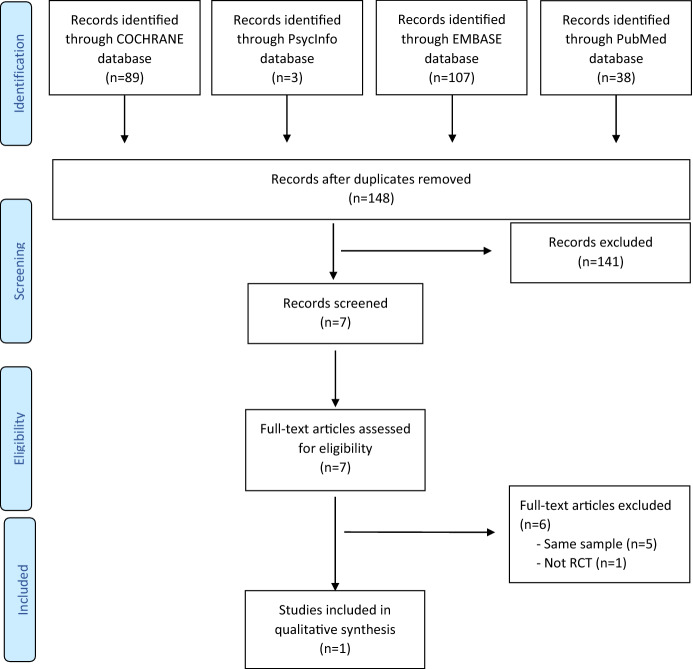
Prisma flow diagram

Two hundred and thirty-seven papers published between 2013 and 2017 were screened (Cochrane = 89, PubMed = 38, Embase = 107, Psycinfo = 3), of which seven were assessed for eligibility. Only one was included in this study (Koenig et al., 2015a, 2015b) since five studies were different analyses conducted on the same sample (Koenig et al., 2014,2015a,2016a, 2016b; Pearce et al., 2015a) and one did not satisfy the definition of a randomized clinical trial (Koenig, 2014b). See Fig. 1 for the PRISMA flowchart which depicted the phases of this systematic review. Other 10 studies were extracted from the prior reviews (up to and including January 2014) (Anderson et al., 2015; Hook et al., 2010; Lim et al., 2014). However, we were unable to retrieve one of the papers for analysis (Johnson et al., 1994). The final sample consisted of 10 studies (Armento, 2011; Bowland et al., 2012; Ebrahimi et al., 2013; Johnson & Ridley, 1992; Koenig et al., 2015b; Pecheur & Edwards, 1984; Propst et al., 1992; Propst, 1980; Razali et al., 2002; Zhang et al., 2002).

We were able to obtain six manuals from the 10 papers included (Bowland et al., 2012; Koenig et al., 2015b; Pecheur & Edwards, 1984; Propst, 1980; Propst et al., 1992; Zhang et al., 2002). Content concerning the techniques in the other four papers was extracted from the description of the intervention reported in the paper describing the clinical trial. See Table 1 for more details on the clinical trials included in this work.

**Table 1:** Summary of included studies

Study	Disorder	n	Intervention	Control	Main results
1. Propst (1980)	Depression	47	Religious imagery (RI)	(1) Nonreligious imagery (2) Therapist contact and self-monitoring (3) Self-monitoring (4) WL	MMPI-total pathology: no difference MMPl-D: RI and (2) was superior to (4) BDI: RI showed lower proportion of depression than (1) or (4)
2. Pecheur and Edwards, (1984)	Depression	21	Religious cognitive behavior modification	(1) Secular cognitive behavior modification (2) WL	BDI, HSRD, VAS, TSCS, HS: no differences between active interventions, both treatments were better than WL
3. Propst et al. (1992)	Depression	66	R-CBT	(1) Standard CBT (2) Pastoral counseling treatment (3) WL	BDI: only R-CBT was superior to WL HRSD: R-CBT and (2) were superior to WL SAS: only R-CBT was superior to WL GSI: no differences
4. Johnson and Ridley, (1992)	Depression	10	Christian rational-emotive therapy (CRET)	(1) Rational-emotive therapy	BDI, ATQ-30: both treatments reduced scores EIV: only CRET reduced irrational believes BID, ATQ-30, EIV: no difference between groups
5. Razali et al. (2002)	Generalized anxiety disorder	200	Supportive psychotherapy + relaxation exercise + BZD + R-CT	Supportive psychotherapy + relaxation exercise + BZD	HAM-A: more rapid improvement in R-CT treatment for religious clients. No differences for non-religious clients
6. Zhang et al. (2002)	Generalized anxiety disorder	143	(1) R-CT (2) BDZ + R-CT	BZD	SCL-90 (1 month): BDZ and BDZ + R-CT superior to R-CT SCL-90 (6 months): BDZ + R-CT and R-CT superior to BDZ CSQ (6 months): R-CT and BDZ + R-CT superior to BDZ Neuroticism (6 months): R-CT superior to BDZ + R-CT and BDZ
7. Armento (2011)	Depression	50	Behavioral activation of religious behaviors (PRA-BA)	No-treatment “support” condition	BAI: PRA-BA superior to control group BDI: PRA-BA superior to control group EROS: PRA-BA superior to control group STAI-T: PRA-BA superior to control group QOLI: no difference between groups
8. Bowland et al. (2012)	Posttraumatic stress disorder	44	R-CBT	Unclear	Depressive symptoms: R-CBT superior to control group Anxiety symptoms: R-CBT superior to control group Somatic symptoms: R-CBT superior to control group
9. Ebrahimi et al. (2013)	Dysthymic disorder	62	R-CBT	(1) CBT (2) Anti-depressive (3) WL	BDI: R-CBT, (1), (2) were better than (3) R-CBT was superior to (2) but not superior to (1) DAS-26: R-CBT were superior to (1), (2), and (3)
10. Koenig et al. (2015b)	Depression and chronic medical illness	132	R-CBT	CBT	BDI: no difference between groups DASI: no difference between groups Response and Remission: no difference between groups R-CBT was better than CBT in more religious clients

*Note. ATQ-30* Automatic Thoughts Questionnaire, *BAI* Beck anxiety inventory, *BDI* Beck depression inventory, *BZD* benzodiazepine; *CSQ* Coping Style Questionnaire; *DAS-26* Dysfunctional Attitudes Scale, *DASI* Duke Activity Status Index, *EIV* Ellis Irrational Values Scale, *EROS* Environmental Reward Observation Scale, *GSI* Global Severity Index, *HAM-A* Hamilton Anxiety Rating Scale, *HRSD* Hamilton Rating Scale for depression, *HS* Hopelessness Scale, *MMPI* Minnesota multiphasic personality inventory, *MMPI-D* Minnesota multiphasic personality inventory—depression, *n* randomized sample size, *QOLI* quality of life inventory, *R-CT* religious cognitive therapy, *SAS* Social Adjustment Scale, *SCL-90* symptom checklist, *STAI-T* state-trait anxiety inventory—trait, *TSCS* Tennessee Self-Concept Scale, *VAS* Visual Analogue Scale to depression, *WL* Waiting list

Depressive disorder was the most frequently studied mental health disorder in these clinical trials (seven studies) (Armento, 2011; Ebrahimi et al., 2013; Johnson & Ridley, 1992; Koenig et al., 2015b; Pecheur & Edwards, 1984; Propst, 1980; Propst et al., 1992). In addition to major depression investigated in five trials, Koenig et al.,(2015a, 2015b) studied R-CBT in chronic medical illness with comorbid depression (Koenig et al., 2015b) and Ebrahimi et al. (2013) focused on Dysthymic Disorder (Ebrahimi et al., 2013). Two other studies assessed R-CBT in Generalized Anxiety Disorder (Razali et al., 2002; Zhang et al., 2002) and one in Posttraumatic Stress Disorder (Bowland et al., 2012). All studies assessed the symptoms of the target disorder. Other investigated outcomes were clinical severity (Pecheur & Edwards, 1984; Propst et al., 1992), response and remission (Koenig et al., 2015a, 2015b), quality of life (Armento, 2011), coping style (Zhang et al., 2002), self-concept and hopelessness (Pecheur & Edwards, 1984), personality and neuroticism (Zhang et al., 2002), trait anxiety (Armento, 2011), automatic thoughts and values (Johnson & Ridley, 1992), social adjustment (Propst et al., 1992), dysfunctional depressive attitudes (Ebrahimi et al., 2013), physical functioning (Koenig et al., 2015b) and environmental reward (Armento, 2011). All these studies were published in the English language.

### Initial Considerations

Eight of the 10 studies supplied an adapted CBT for religious clients. However, one study randomized a secular treatment and a religiously adapted treatment for religious and non-religious clients (Razali et al., 2002), while another study included two atheist clients (one in the R-CBT group) (Armento, 2011). One of these studies reported a positive outcome only for religious clients (Razali et al., 2002). Seven of the 10 protocols explicitly instructed therapists to respect clients’ religious beliefs and opposed attempts to proselytize (Armento, 2011; Bowland et al., 2012; Koenig et al., 2015a, 2015b; Propst, 1980; Propst et al., 1992; Razali et al., 2002; Zhang et al., 2002). The goal of R-CBT is to use the client’s own religious beliefs, values, teachings, and practices to help resolve mental health issues. Therapists do not need to share the beliefs of their clients to administer spiritually adapted protocols.

Except for Bowland et al. (2012) and Propst (1980), all protocols were offered individually rather than in groups. In general, the duration of the sessions varied from 45 to 90 min, offered on a weekly basis, and the number of sessions varied from eight to 12 (Bowland et al., 2012; Ebrahimi et al., 2013; Koenig et al., 2015b; Pecheur & Edwards, 1984; Propst, 1980; Propst et al., 1992). Some protocols were briefer, consisting of two (Armento, 2011) to six sessions (Johnson & Ridley, 1992) over two (Armento, 2011) to three weeks (Johnson & Ridley, 1992). The protocol advocated by Zhang et al. (2002) consisted of one month of weekly sessions and five months of biweekly sessions. One study offered the intervention by phone and online (Koenig et al., 2015a, 2015b).

Below, we explore the adapted interventions from the perspective of the clinical practice. We classified the interventions as *Adapted Assessment, Adapted Psychoeducation, Adapted Motivational Strategies, Adapted Behavioral Interventions, Life Values and Adapted Cognitive Interventions.* We chose this classification system as we believe it provides clinicians with information about which adaptations have supporting research in order to guide the adaptations clinicians might use in their practices. The information can also be used by researchers who want to study new protocols or components of R-CBT. See Table 2 for a summary of the interventions.

**Table 2:** Summary of interventions

	Techniques	Description
Initial therapy session	Psychoeducation about symptoms and mental health disorders Psychoeducation about CBT model	Discussion about religious theories for the causation of symptoms and disorders. Information provided about the impact of R/S on mental health Explanation about the relationship between thoughts, behaviors, and emotions using R/S content
Motivational strategies		Use R/S content to motivate clients to engage in treatment
	Religious activities	Explicitly encourage private religious activities (e.g., praying, reading religious texts, meditating) and religious community activities (e.g., religious services, engaging in charity), and R/S activities as homework tasks. These activities are used for behavioral activation
Behavioral intervention	Coping strategies	Encouraging clients to cope using positive R/S strategies (e.g., secure relationship with God, sense of spiritual connectivity with others) and reducing the use of negative R/S coping strategies (e.g., viewing God as punishing or abandoning them, being unable to forgive)
Life values		Motivating clients to act according to their R/S values (e.g., forgiveness, generosity, altruism, compassion)
Cognitive intervention	Cognitive restructuring Religious imagery modification	Modifying distorted automatic thoughts and beliefs using R/S content Combining cognitive restructuring with systematic desensitization. Clients are encouraged to imagine a depressive image while also imagining themselves coping using a R/S perspective

*Note. R/S* religious or spiritual

### Adapted Assessment

The CBT assessment aims to collect information about the client to develop a formulation of the case and create a cognitive concept about the client, to diagnose disorders, explain the cognitive and behavioral model, explain the treatment, and create a treatment plan (Beck et al., 2013). A spiritually adapted assessment adds to a standard clinical assessment of the religious/spiritual history of a client to better understand the relationship between a client’s religious beliefs, practices, and values and the problem presented. With this objective, one study invited clients to clarify their religious/spiritual experiences as well as their involvement in religious activities in the community and in private (Armento, 2011). If clients were atheist or agnostic, they were invited to describe this experience too (Armento, 2011). This protocol also encourages the therapist to consult specific literature about a client’s religion (Armento, 2011). The manuals produced by Koenig et al., (2015a, 2015b) encourage therapists to identify the religious language and symbols used by the client. In the first section of the Bowland et al. manual, individuals discuss their understanding of spirituality and their religious and spiritual experiences, both positive and negative. The therapists define the experiential dimensions of spirituality and religion as synonymous, but state that religion is related to an institution (Bowland et al. manual). Therapists also define religious and spiritual coping, both positive and negative, so that individuals can identify their own positive spiritual resources (Bowland et al. manual). There follows an example of a question about experiences of religiousness (Bowland et al. manual, p.6):

What aspect of your spiritual life has been most important to you? What has been most helpful? Least helpful?

### Adapted Psychoeducation

In each of the protocols, the initial sessions are designed to evaluate the client and provide psychoeducation about the cognitive behavioral model and the disorder, as well as to introduce the treatment rationale. Psychoeducation is an essential part of CBT since the objective of CBT is to help the clients develop abilities so they can become their own therapist, to use these abilities to modify cognition, emotions and behavior (Wright et al., 2008). Thus, the rationale for integrating religiousness into CBT can also be provided in these initial sessions. Some protocols provide explicit information to clients about the benefits of religion in reducing anxiety and depressive symptoms (Armento, 2011; Zhang et al., 2002). For example, Armento's manual instructs the therapist to provide information about the benefits of religiosity through studies showing the association between religious activity and lower levels of depression (Armento, 2011). Also, the Zhang et al. manual presents the client with research evidence involving studies assessing Taoist principles, for alleviating symptoms of anxiety and worry and improving the quality of life.

In addition to providing the customary CBT psychoeducation, some protocols also discuss religious theories for the formation of mental health symptoms (Bowland et al., 2012; Ebrahimi et al., 2013; Pecheur & Edwards, 1984; Propst, 1980; Propst et al., 1992; Razali et al., 2002). Pecheur and Edwards (1984) reviewed biblical teachings about the self, the world and the future with Christian clients suffering from depression. The relationship between mental health and religiousness, the use of religiousness for coping (Armento, 2011), the role of prayer and faith (Koenig et al., 2015b), and the value of spiritual coping (Bowland et al., 2012) were also introduced by some manuals.

The client’s beliefs were also used to explain the therapeutic rationale (Ebrahimi et al., 2013; Koenig et al., 2015a, 2015b; Pecheur, 1980; Propst, 1980; Propst et al., 1992). Many religions teach that our thoughts, behaviors, and emotions are interconnected and therefore influence each other. See below an example of the relationship between cognition, feelings, and behavior:

The ancient Greek word “metanoia” literally means to “change your mind” or “change how you think,” which the Bible translated as “repent.” Thus, changing the way we think (i.e., repenting in Christian language) is a biblical concept (Matthew 4:17) (Pearce et al., 2015a, 2015b).

The importance of becoming an observer of one's own thoughts and the idea that thought modification is a therapeutic mechanism are concepts found in several world religions’ sacred texts. An example from Christianity (Pecheur, 1980):

And be not conformed to this world: but be ye transformed by the renewing of your mind, that ye may prove what is that good, and acceptable, and perfect, will of God (Romans 12:2).

See Table 2 for a summary of adapted initial interventions.

### Adapted Motivational Strategies

Specifically, motivational interviewing is a tool to help clients recognize their problems and do something to modify them. It is especially useful for clients who are ambivalent about change (Miller & Rollnick, 2001). Thus, religious content can be used to motivate clients to better engage with their treatment. Specific teachings of many religious traditions, such as “life has a purpose” and “God expects individuals to improve themselves,” can motivate religious clients to strongly engage with their treatment. Propst’s manual, for example, uses specific examples of Christ to motivate depressive clients to develop positive activities and to adopt more assertive behavior, social skills, and goal setting (Propst, 1980; Propst et al., 1992). See Table 2 for a summary of adapted, motivational strategies.

The Koenig et al. manual provided two examples of how therapists can use a client’s religious beliefs to help motivate them to engage with their treatment (Pearce et al., 2015a, 2015b):

For monotheistic clients: “…therapists emphasize that there are many things God asks people to do that they don’t necessarily feel like doing. People do these things because they believe that a loving God would only ask them to do things that are ultimately for their benefit.” (Pearce et al., 2015b, p.8).

For Jewish clients: “An effective way to change our mood is to engage in pleasant activities. One of the first steps in changing our perceptions and negative thoughts is to begin to see the good things in our environment and to make some of them a part of our daily activity. This idea is consistent with Torah thought.” (Pearce et al., 2015b, p.8).

### Adapted Behavioral Interventions

#### Religious Activities

Religious activities can be performed privately or in a faith community. Most protocols explicitly suggested the use of private religious activities, such as praying for self and others, reading religious texts and meditating on religious passages (Armento, 2011; Bowland et al., 2012; Ebrahimi et al., 2013; Johnson & Ridley, 1992; Koenig et al., 2015b; Razali et al., 2002). Faith community activities, such as seeking support by joining a spiritual group, spending time with members of one’s faith community, participating in study groups and engaging in charitable activities, were also encouraged (Bowland et al., 2012; Koenig et al., 2015b). See Table 2 for a summary of adapted behavioral interventions.

Armento (2011) developed a Behavioral Activation protocol by fostering religious behaviors. Behavioral Activation consists of a simple procedure in which the therapist engages the clients in a process of change and encourages activity﻿ (Wright et al., 2008). It is essential for most depressive clients (Beck et al., 2013), and usually, it is already implemented in the initial sessions﻿ (Wright et al., 2008). Armento’s protocol comprises two sessions. In the first session, clients were helped to better understand their symptoms and how their religious beliefs impacted their mental health (Armento, 2011). This protocol used the *Serenity Prayer* to engage clients to change what they can change and to accept what they cannot:

God, give me grace to accept with serenity the things that cannot be changed, Courage to change the things which should be changed, and the Wisdom to distinguish the one from the other. Living one day at a time, Enjoying one moment at a time, Accepting hardship as a pathway to peace, Taking, as Jesus did, This sinful world as it is, Not as I would have it, Trusting that You will make all things right, If I surrender to Your will, So that I may be reasonably happy in this life, And supremely happy with You forever in the next. Amen (Niebuhr, 1951).

Then, they began the process of religious activation by adding religious activities to behavioral activation (Armento, 2011). As mentioned above, this protocol used the “Serenity Prayer” to inspire clients to think about “acceptance versus change” and to motivate them to engage with their treatment (Armento, 2011). Ways to be more active in the community and engage more in private religious practices were explored. Clients were then invited to engage in these activities for the next two weeks (Armento, 2011). After the first week, clients received a phone call to check on their progress and to solve problems concerning the completion of their homework (Armento, 2011). In the second and final session, the experience and progress of the client were discussed (Armento, 2011). Here is the list of suggested activities in this protocol (Armento, 2011):

Community Activities*:* “attend a sacred liturgy, minister at a religious service or function, attend a bible study, attend a religious social function, attend a young adults’ group, speak with a religious leader, volunteer at a special event.”

Private Activities: “go to a peaceful place and pray privately, read the Bible, read a devotional book, private prayer or meditation at home, listen to prayerful music, go for a walk or a hike, learn more about your faith.”

#### Coping Strategies

Some protocols also focus on R/S coping strategies. The Zhang et al. manual focuses on coping strategies to help clients with generalized anxiety disorder (GAD) to act in accordance with their values, needs, and desires. It also teaches clients how to deal with worry and distress and to change the way one copes with life events based on traditional Chinese philosophical principles. To teach clients how to use religious coping strategies, Zhang et al. (2002) developed an “ABCDE” method of Chinese Taoist Cognitive Psychotherapy. The A represents “Actual stress factors”; B, “Belief and value system”; C, “Conflict and coping style”; D, “Doctrine direction and practice”; and E, “Effect evaluation and reinforcement.” In step “B,” clients classified their beliefs and values according to 10 identified needs: money, freedom, love, position, health, power, fame, friendship, family, and enjoyment. In step “C,” therapists helped clients assess their conflicts and coping styles using a list of commonly used coping styles in China: “*(1) to suppress or deny, (2) to express oneself openly, (3) to sublimate, (4) to abuse substances, (5) to vent, (6) to punish oneself, (7) to withdraw and console oneself, and (8) to engage in recreational activities”* (Zhang et al., 2002). In step “D,” clients were exposed to Taoist teachings (Zhang et al., 2002):

Benefiting without hurting others and acting without striving,

Restricting selfish desires, learning to be content, and knowing how to let go,

Being in harmony with others and being humble, using softness to defeat hardness, and

Person should maintain tranquility, act less, and follow the laws of nature.

Firstly, therapists explain how the principles of Taoism can help clients with GAD. Then, specific aspects of Taoist doctrine are taught and discussed concerning how to overcome GAD. Afterwards, the client is provided with psychoeducation about the rationale of the adapted treatment and about Confucianism and Taoist philosophy, including the “32-character Taoist formula”, which consists of eight principles. Clients are encouraged to discuss, reflect and apply each of these principles as a way of coping with anxiety. To reflect on these principles, chapters from the Daodejing are provided and explained. To assist in applying these principles in daily life, a CBT-like Thought Record is completed and alternate ways of coping based on Taoist principles are discussed. Many of the sessions are designed to assist clients in applying the Taoist principles to cope better with worry and stress (Zhang’s manual).

Despite individuals generally using spirituality in moments of crisis to *"comprehend the seemingly incomprehensible and to manage the seemingly unmanageable"* (Pargament, 2011), sometimes the spiritual dimension can also be a field of conflict and suffering (Pargament, 2011). With the intention of modifying the use of negative religious coping strategies, Bowland et al. (2012) helped clients restructure their interpretations of traumatic experiences. They provided psychoeducation about positive coping strategies and negative religious coping strategies (Bowland et al., 2012). Spiritual histories of traumatized women were reviewed and alternative interpretations were suggested based on the Christian scriptures (Bowland et al., 2012). In Session 3 of this manual (Bowland et al., 2012), clients’ religious coping strategies are identified, both positive and negative, and positive coping strategies are encouraged. Also, steps to deal with spiritual struggles are actively developed by each group member.

In addition, emotions and feelings that arise from a traumatic event, like anger, fear, powerlessness, shame, guilt, loneliness, or despair, can be difficult to accept in some religious traditions. As such, each of these feelings is discussed in sessions 4 to 8 and positive religious coping strategies to deal with these emotions are explored and encouraged.

See Table 2 for a summary of adapted coping interventions.

### Life Values

Most religions promote values like forgiveness, generosity, altruism, and compassion. Research shows that acting in accordance with one’s values is important for mental health (VanderWeele, 2017). Several of the manuals explore the client’s values.

Bowland et al. (2012) adapted a pre-existing protocol (Trauma Recovery and Empowerment Model) for traumatized Christian clients, applied in a group (Bowland et al., 2012). In Session 2, qualities like discernment, forgiveness, and trust were discussed and clients identified these values as something that they either had or desired more of in their lives (Bowland et al., 2012). Also, the literature concerning forgiveness and health was explicitly discussed during the treatment, to encourage forgiveness (Bowland et al., 2012). To assess these spiritual “virtues,” a list of spiritual gifts was provided (Bowland’s manual, p.19):

Acceptance, Accountability, Believing, Blessing, Centeredness, Clarity, Commitment, Compassion, Connection, Contentment, Courage, Detachment, Discernment, Discipline, Faith, Forgiveness, Gratitude, Guidance, Healing, Joy, Kindness, Leadership, Love, Meditative, Mercifulness, Miraculous, Patience, Peacefulness, Praise, Prayerfulness, Prosperity, Purpose, Serenity, Teaching, Tolerance, Trust, Understanding, Wisdom.

Subsequently, the group was encouraged to develop its own list and to assess how these virtues are present in their daily lives (Bowland’s manual). In the last three sessions, forgiveness, hope, vision, and healing were assessed, since religiousness can help trauma survivors recover and develop these values. In terms of forgiveness, for example, clients were invited to define forgiveness and to distinguish it from the idea of “letting go,” recognizing it can be difficult for survivors to forgive the aggressor. The Bowland et al. manual also describes the work as a “spiritual journey” where trauma survivors overcome their trauma for the long term. Religious resources for healing are assessed and how to use these resources and create a healing space is encouraged. The group ended with a reflection such as the “Prayer of Loving Kindness” (Bowland’s manual, p.18):

May you dwell in the heart

May you be free from suffering

May you be healed

May you be at Peace.

The Zhang et al. manual also uses clients’ values as an important part of the therapy by introducing the concept of values, assessing the client’s values and determining to what extent they are living by these values (Zhang et al., 2002). The client’s values are assessed through open-ended questions and by using the Valued Living Questionnaire, both of which are used to contrast a client’s stated values with how much he/she has complied with them. Afterwards, clients examine conflicts between what they value and how they are behaving and are encouraged to develop strategies to act in greater compliance with their stated values.

Koenig et al., (2015a, 2015b) also included in their protocol values such as forgiveness, meaning and purpose, gratefulness, gratitude, altruism, and social engagement and did so based on the specific religious teachings of the clients (Koenig et al., 2015b). They explicitly encouraged clients to practice exercises in gratitude and to act with altruism and generosity toward others (Koenig et al., 2015b; Koenig, 2014a).

### Adapted Cognitive Interventions

#### Restructuring Negative Automatic Thoughts and Irrational Beliefs

The cognitive behavioral model posits that the cognition associated with the interpretation of a situation leads to emotions, behaviors, and physiological responses (Beck et al., 2013). Clients with mental disorders tend to have distorted cognition about neutral or positive situations (Beck et al., 2013). So, we must examine and correct these cognitions to achieve improvement in mental symptoms (Beck et al., 2013). In general, cognitive restructuring involves firstly identifying distorted automatic thoughts (i.e., the flow of thoughts common to all of us) and then modifying them using cognitive restructuring techniques (Beck et al., 2013). In the adapted protocols, therapists use the client’s religious rationales and values as resources for cognitive restructuring (Ebrahimi et al., 2013; Koenig et al., 2015b; Pecheur, 1980; Pecheur & Edwards, 1984; Propst, 1980; Propst et al., 1992; Razali et al., 2002). In other words, automatic thoughts are identified and then changed using religious values, teachings, and beliefs. For example, protocols adapted for Christians use biblical scriptures (Koenig et al., 2015b; Propst et al., 1992), while Muslim protocols use the Holy Qur’an and Hadith (Koenig et al., 2015b; Razali et al., 2002). Some examples of verses from the Bible that can be used in the cognitive restructuring of Christian clients suffering from depression due to negative views of the self are given below:

Look at the birds of the air, for they neither sow nor reap nor gather into barns; yet your heavenly Father feeds them. Are you not of more value than they? (Matthew 6:26) (Pecheur 1980).

Naturally, any scriptures or passages from holy texts need to be taken specifically from the clients' religious tradition and need to resonate with their particular system of belief.

Koenig et al., (2015a, 2015b) used an adapted instrument for cognitive restructuring called the “ABCD(R)E” thought log, based on Albert Ellis’s method for cognitive restructuring. In their protocol, they added step “R,” which stands for religious beliefs and resources (Koenig et al., 2015b; Pearce et al., 2015a, 2015b). In step R, clients are invited to challenge their automatic thoughts with their own religious beliefs, values, and teachings (Koenig et al., 2015b; Pearce et al., 2015a, 2015b):

How can your view of God, your religious/spiritual worldview, religious writings, spiritual wisdom, and other sources provide evidence that challenge your automatic negative beliefs and beliefs that you can’t cope? (Koenig et al., 2015b; Pearce et al., 2015a, 2015b).

Below is an example, reported by this manual, of religious cognitive restructuring for some depressive “should and must” statements:

Buddhism Religion: “When we use the word “should,” there is generally little room for self-acceptance or flexibility. The Buddha taught that guidelines for our own behavior can be important, but that these need to come from a place of caring and love for others, and from a place of higher wisdom and caring for ourselves. Such wisdom may reflect the recognition that situations are often complex and that a single mode of action or behavior is not even desirable or useful. (Koenig et al., 2015b; Pearce et al., 2015a, 2015b).

Another study integrated Christian principles into Rational-Emotive Therapy (Johnson & Ridley, 1992). In Johnson and Ridley’s manual, clients were encouraged to question four categories of depressive irrational beliefs (catastrophizing statements; should/ought/must; human worth assessment; and need statements) using biblical scripture rather than secular arguments (Johnson & Ridley, 1992). Firstly, irrational beliefs are identified and labeled, as well as their triggers and consequences (Johnson & Ridley, 1992). Next, clients are instructed on how these irrational beliefs are responsible for their emotions. They are then taught how to contest their irrational beliefs based on teachings from the Bible (Johnson & Ridley, 1992). See below an example of a “must” statement:

I must be thoroughly competent, adequate, and achieving in all possible respects if I am to consider myself worthwhile,” would become: “All of us have become like one who is unclean and all our righteous acts are like filthy rags (Isaiah 64:4) (Johnson & Ridley, 1992).

#### Religious Imagery Modification

Many automatic thoughts are experienced through images or mental pictures (Beck et al., 2013). As this is achieved by means of automatic thoughts, we can identify and correct these images (Beck et al., 2013). The Religious Imagery Modification is a cognitive technique that helps to change depressive images using religious content and combines cognitive restructuring with systematic desensitization techniques (i.e., clients are invited to restructure their experiences using religiously based imagery) (Propst, 1980; Propst et al., 1992). Systematic desensitization involves exposure to the feared situations in a hierarchical and gradual way (Wright et al., 2008). In this adapted technique, clients imagine a depressive scene and the therapist uses this scene to help clients become more aware of their automatic thoughts and distortions. Clients then meditate on an image of Christ, with the intention of imagining themselves effectively coping with depressive thoughts (Propst, 1980; Propst et al., 1992).

Rather than using imagery, Koenig et al., (2015a, 2015b) encouraged clients to practice contemplative prayer, meditating on a specific religious passage to help clients better manage negative thinking patterns (Koenig et al., 2015b; Pearce et al., 2015a, 2015b). They instructed clients to choose a scripture (and also provided a sacred passage from the client’s holy scriptures related to the session content that the client could use during contemplative prayer) (Koenig et al., 2015b; Pearce et al., 2015a, 2015b). They were then asked to follow these instructions:

Savor each phrase. What word phrase or idea speaks to you? Read the passage again. Where does this passage touch your life? What do you see, hear, touch, or remember? Read the passage a third time. Listen quietly. Note insights, reflections, and personal response to the reading in your journal. Follow the steps in order or go back and forth between them as you feel moved. Finish by waiting for a few moments in silence. (Pearce et al., 2015b, p. 20).

In Pecheur’s protocol, clients were asked to recall depressive thoughts and feelings (Pecheur & Edwards, 1984). Then, they were given lists of religious coping statements and images (Pecheur & Edwards, 1984) using Beck's cognitive triad of depression (i.e., distorted and pessimistic concept of self, world, and future) (Beck, 1976). Clients were invited to replace their negative thoughts and images with images such as visualizing Christ going with them into a difficult situation. For example: *"I can visualize Christ going with me into that difficult situation in the future as I try to cope"* (Propst, 1980).

#### Restructuring Negative Automatic Thoughts and Irrational Beliefs Related to Faith

In addition to restructuring distorted cognitions related to the client’s mental health disorder, some studies encouraged clients to restructure negative thoughts and beliefs related to their faith (i.e., spiritual struggles, such as feeling that God is punishing them). More specifically, studies encouraged clients to use religious positive coping strategies (e.g., spiritual beliefs that give meaning to existential questions and suffering, a secure relationship with a forgiving and acceptant God, a sense of spiritual connection with others) and discouraged the use of negative religious coping strategies (e.g., viewing God as punishing or abandoning them, being unable to forgive) (Razali et al., 2002; Zhang et al., 2002). See Table 2 for a summary of adapted cognitive interventions.

## Discussion

To our knowledge, this is the first review paper to identify and describe R-CBT techniques that have shown efficacy in randomized clinical trials in clients with mental health disorders. The final sample for this review consisted of 10 studies. In general, the protocols combined different cognitive and behavioral techniques with religious content to help clients use their own religious beliefs and practices, rather than those of the therapist. The most common spiritually integrated CBT techniques were: using religious content to help restructure automatic thoughts or distorted beliefs (Ebrahimi et al., 2013; Johnson & Ridley, 1992; Koenig et al., 2015b; Propst et al., 1992; Razali et al., 2002; Zhang et al., 2002) and encouraging private religious activities and involvement in religious community activities (Armento, 2011; Bowland et al., 2012; Ebrahimi et al., 2013; Johnson & Ridley, 1992; Koenig et al., 2015b; Razali et al., 2002) as both techniques are described in six out of the 10 studies.

Concerning the efficacy in clients with depressive disorder, R-CBT was superior to the waiting list for reducing symptoms (Ebrahimi et al., 2013; Pecheur & Edwards, 1984; Propst, 1980; Propst et al., 1992). Moreover, studies found that R-CBT was superior to the comparison group (Armento, 2011) and pharmacotherapy (Ebrahimi et al., 2013). When compared to secular CBT, the results are inconclusive. Some studies found that R-CBT was no more effective than secular CBT (Ebrahimi et al., 2013; Pearce et al., 2015a, 2015b; Pecheur & Edwards, 1984). Propst et al. found that R-CBT was only superior to the waiting list for reducing depressive symptoms (Propst et al., 1992), but not secular CBT, and only the religious imagery group (but not secular CBT) showed a lower proportion of depressed individuals than the waiting list (Propst et al., 1992). Interestingly, Johnson and Ridley (1992) found that only R-CBT reduced irrational beliefs (but not secular rational-emotive therapy).

In relation to anxiety spectrum disorders, two studies assessed R-CBT in Generalized Anxiety Disorder (Razali et al., 2002; Zhang et al., 2002), and one in Posttraumatic Stress Disorder (Bowland et al., 2012). Meanwhile, Razali et al. (2002) compared supportive psychotherapy, relaxation exercise, benzodiazepines, and R-CBT with supportive psychotherapy, relaxation exercise, and benzodiazepines, independently, and found that religious clients receiving R-CBT achieved a more rapid improvement in symptoms of anxiety, but there were no differences in the follow-up and no differences for non-religious clients. Zhang et al. (2002) compared R-CBT and R-CBT plus benzodiazepines with only benzodiazepines and reported that those who received R-CBT (with or without benzodiazepines) showed better improvement in their symptoms when compared to those who only received benzodiazepines in the follow-up (6 months). Also, R-CBT was superior to benzodiazepines for improving neuroticism, an important trait associated with generalized anxiety disorder. Finally, in Bowland et al. (2012), R-CBT was superior to the control group for reducing depressive anxiety and somatic symptoms in women survivors of trauma. However, the nature of the control group is unclear.

It is important that therapists know how to use spiritually integrated therapies. The vast majority of the world's population (84%) reports some religious affiliation and most clients want their therapist to address religiousness in their psychotherapeutic process (Post & Wade, 2009); moreover, religion has an impact on mental health (Koenig, 2009; Moreira-Almeida et al., 2014). Considering these issues, it is unethical for clinicians not to address religiousness in treatment, to paraphrase Rosmarin in his book about addressing spirituality and religion in the practice of CBT (Rosmarin, 2018). A religion-adapted CBT is needed because it can make religious patients feel more at ease with psychotherapy and it helps the therapist to identify and mobilize R/S resources that the patient or his/her environment may have and which may ultimately help in the psychotherapeutic process. In addition, R-CBT techniques should not be used in a full, standardized protocol, but they can and should be incorporated into all kinds of psychotherapeutic approaches. However, there is a gap between scientific knowledge and clinical practice, and one of the obstacles attributed to this gap is the lack of information on how to address religiosity in practice. Thus, describing psychotherapeutic techniques is fundamental to reducing the "how to do" gap.

R-CBT allows the therapist to include the client's religious cognitions and practices to reduce symptoms and improve function. In addition, maladaptive aspects of the client's religiousness that contribute to suffering may be the focus of treatment. As Pargament asserted: *“when people walk into the therapist’s office, they don’t leave their spirituality behind in the waiting room”* (Pargament, 2011). So, addressing religiosity should be dealt with like other aspects of life such as culture, finances, race, and sexual orientation. Besides, the inclusion of these aspects demonstrates respect for the culture to which our client is devoted.

However, the adaptation must be client-centered, i.e., not just the client's religion, but also the way he/she understands and practices the religion should be considered. Moreover, therapists should take care of ethical aspects and should not attempt to proselytize. Again, the content used in the therapy session must be related to the client's faith. The therapist's beliefs should not be considered, nor his/her materialistic beliefs. The role of the therapist should not be confused with pastoral counseling. Finally, Rosmarin also points out that the integration of religiosity into CBT must be determined empirically. Thus, we should evaluate its results for each client throughout the treatment (Rosmarin, 2018).

There are a few limitations that should be noted in this review. Like studies on conventional CBT, one important limitation is that we do not know which strategies are the "active ingredients" that resulted in a change in symptoms, since the studies evaluated protocols comprising diverse techniques. Secondly, most of the studies included did not report if they checked for fidelity to cognitive behavioral techniques. The term CBT was perhaps used loosely in some studies while other studies seemed to produce a better fit to the third wave of CBT, i.e., a group of interventions that focus on the process of acceptance, mindfulness, attention, and values (Hayes et al., 2011). A third limitation is that many of the studies did not report the complete protocols and/or provide examples, and we did not have access to all the treatment manuals. However, we did have access to six protocols and retrieved information from the techniques in the methodological descriptions or appendices of the other studies.

Importantly, most studies were performed with Christian clients. Thus, many examples provided are relevant for Christian clients. So, studies are lacking for different cultures, mainly Eastern cultures. However, despite the diversity of religions, many concepts and practices are similar. Thus, these adapted techniques can provide the basis for the treatment of clients of different religions (Rosmarin, 2018) and can be used as an example for new psychotherapeutic adaptations for different cultures. Finally, we only included studies published in English, Portuguese and Spanish, although only one study was excluded based on the language criterion.

Despite these limitations, this study is the first to describe adapted interventions based on a careful review of the literature, on how to integrate clients' beliefs and practices into CBT in a culturally sensitive way. The compilation of this treatment information makes for easy access for busy therapists who want to implement empirically based adaptations of CBT for their religious clients. Religious-adapted psychotherapy is still in its early stages of development. However, this study does represent a step toward advancing spiritually integrated psychotherapy as it aims to help therapists develop skills in religious-adapted CBT in a more formal way (Pargament, 2011). Also, it is important to note that personal religiousness and spirituality do not prepare therapists for this kind of intervention and therapists should be instructed not to attempt to proselytize, imposing their view of religion on clients (Pargament, 2011).

We hope that this review of spiritually adapted CBT protocols provides practical information for clinicians to use with their religious clients and provides helpful information for future research on the components of these approaches.

## Conclusion

Various adapted tools can be integrated into CBT to better tailor the treatment to clients' cultural values and resources. We described these techniques using the best evidence available from reviews of the manuals used in clinical trials comparing R-CBT to control conditions in psychiatric settings. The inclusion of religious issues in psychotherapy is not only feasible but also ethical and must be implemented according to the wishes of each patient.
